# Preoperative and Intraoperative Identification of Sentinel Lymph Nodes in Melanoma Surgery

**DOI:** 10.3390/cancers16152767

**Published:** 2024-08-05

**Authors:** Stanley P. Leong, Mehdi Nosrati, Max C. Wu, Donald M. Torre, Ted F. Bartley, Kevin B. Kim, Christopher Soon, John Moretto, Mohammed Kashani-Sabet

**Affiliations:** 1 Center for Melanoma Research and Treatment, California Pacific Medical Center Research Institute, San Francisco, CA 94115, USA; mehdi.nosrati@sutterhealth.org (M.N.); mohammed.kashani-sabet@sutterhealth.org (M.K.-S.); 2 University of California School of Medicine San Francisco, San Francisco, CA 94158, USA; 3 Sentinel Node Oncology Foundation, Novato, CA 94947, USA; 4 Department of Nuclear Medicine, California Pacific Medical Center, San Francisco, CA 94107, USA; max.wu@sutterhealth.org (M.C.W.); donald.torre@sutterhealth.org (D.M.T.); ted.bartley@sutterhealth.org (T.F.B.); 5 Department of Medical Oncology, California Pacific Medical Center, San Francisco, CA 94107, USA; kevin.kim2@sutterhealth.org; 6 Department of Pathology, California Pacific Medical Center, San Francisco, CA 94107, USA; christopher.soon@sutterhealth.org (C.S.); john.moretto@sutterhealth.org (J.M.)

**Keywords:** melanoma, sentinel, lymph nodes, identification

## Abstract

**Simple Summary:**

A sentinel lymph node (SLN) biopsy is recommended for melanoma patients with a Breslow thickness of at least 1 mm. This procedure is crucial for staging and determining the extent of melanoma metastasis. To improve the accuracy of SLN identification and reduce false negatives, the use of the Sentinella gamma camera is introduced intraoperatively. This tool enhances the identification rate of SLNs as compared to the traditional gamma hand-held probe. Surgeons perform the ex vivo dissection of the resected SLN clusters to differentiate sentinel from non-sentinel lymph nodes, which are then examined pathologically. At the Center for Melanoma Research and Treatment at the California Pacific Medical Center, a comprehensive multidisciplinary approach is implemented to discuss the diagnosis and treatment of melanoma patients at the weekly Melanoma Tumor Board. Pathologists evaluate surgical specimens to ensure precise diagnosis. Radiologists discuss imaging studies. A consensus-driven approach is taken by dermatologists, surgeons, medical oncologists, and radiation oncologists at the Melanoma Tumor Board to tailor treatment plans for each melanoma patient. This multi-disciplinary program ensures personalized and high-quality care for melanoma patients and can serve as a model for treating other types of cancer.

**Abstract:**

According to the American Joint Commission on Cancer (AJCC) 8th edition guidelines, SLN biopsy is recommended for primary melanomas with a Breslow thickness of at least 1 mm. Additionally, the National Comprehensive Cancer Network (NCCN) recommends that a SLN biopsy may be considered for melanoma patients with T1b lesions, which are 0.8–1 mm thick or less than 0.8 mm thick with ulceration. It can also be considered for T1a lesions that are less than 0.8 mm thick but have other adverse features, such as a high mitotic rate, lymphovascular invasion, or a positive deep margin. To reduce the false negative rate of melanoma SLN biopsy, we have introduced the intraoperative use of Sentinella, a gamma camera, to enhance the identification rate of SLNs beyond that of the traditional gamma hand-held probe. At the Center for Melanoma Research and Treatment at the California Pacific Medical Center, a multidisciplinary approach has been established to treat melanoma patients when the diagnosis of primary melanoma is made with a referral to our melanoma center. This comprehensive approach at the melanoma tumor board, including the efforts of pathologists, radiologists, dermatologists, surgical, medical and radiation oncologists, results in a consensus to deliver personalized and high-quality care for our melanoma patients. This multidisciplinary program for the management of melanoma can be duplicated for other types of cancer. This article consists of current knowledge to document the published methods of identification of sentinel lymph nodes. In addition, we have included new data as developed in our melanoma center as newly published materials in this article to demonstrate the utility of these methods in melanoma sentinel lymph node surgery. Informed consent has been waived by our IRB regarding the acquisition of clinical data as presented in this study.

## 1. Introduction 

The worldwide incidence of melanoma is rising [1]. A SLN biopsy is indicated for patients with primary melanoma with T1b lesions and beyond based on the recent classifications of the 8th AJCC edition [2]. The status of the SLN is the strongest predictor of melanoma-specific survival by the Multicenter Selective Lymphadenectomy Trial-1 (MSLT-I) [3]. Although MSLT-II [4] has again shown that SLN status is a strong predictor of melanoma-specific survival, the therapeutic benefit of completion lymph node dissection (CLND) has not been demonstrated. Other studies have also shown the utility of SLNs to prognosticate melanoma survival [5,6,7]. Based on the level I evidence from these prospective randomized clinical trials, patients with a negative SLN biopsy will be spared a CLND, and in patients with low tumor burden in the SLN(s), CLND may not be necessary [4]. The SLN biopsy is a less morbid procedure with a complication rate of about 5% [8] when compared with the CLND complication rate, which is as high as over 50% [9]. The importance of the SLN approach is that if the SLN biopsy is negative, radical lymphadenectomy and its associated higher risks can be avoided.

The SLN biopsy method for staging regional lymph nodes and avoiding traditional radical lymphadenectomy was developed through the pioneering work of two SLN surgeons, Cabanas [10], with the penile cancer model focusing on anatomical SLN localization, and Morton [11], who adopted a physiology-based approach centered on lymphatic flow from the primary site to the SLN in melanoma. Subsequent clinical studies in melanoma [4] and breast cancer [12] have firmly established the SLN biopsy as a reliable method for assessing regional lymph node areas. Initially, blue dye or isosulfan blue (Lymphazurin™) was used to detect melanoma SLN [11], but a key advance was the incorporation of radioactive tracers [13,14] for SLN detection. Technetium-99m sulfur colloid is popular because it achieves two key goals: (1) it defines the preoperative drainage pattern from primary cancer, such as melanoma [14] or breast cancer [15], to the regional lymph node basin, and (2) it helps to localize the SLN intraoperatively using a gamma probe [13].

A preoperative lymphoscintigraphy is critical to identify the SLN basin of primary melanoma, which can originate anywhere in the body. Single or multiple node basins may be detected. For example, melanomas in the lower extremities may drain into the popliteal, inguinofemoral, and pelvic nodal basins. In contrast, lesions in the upper extremities may go to the epitrochlear and axillary basins. Melanoma of the trunk may have multiple drainage points, including the lower neck, axilla, and groin. In contrast, primary breast cancer usually travels to the ipsilateral axilla. It is critical to perform preoperative lymphoscintigraphy to map the specific lymph node basins draining from the melanoma site [16].

Morton et al. [11] first described SLN localization in melanoma using vital dye such as Lymphazurin (isosulfan blue) to permit intraoperative mapping. Using blue dye alone, rates of identifying SLN ranged from 52% to 95% [17]. When radiotracer was used by the late 1990s, the SLN identification rate rose to 98% [18]. Using the 10% rule [19] in a retrospective study with 1152 melanoma patients undergoing SLN biopsy by Liu et al. [20], the SLN positivity rate showed no difference between the patients with radiocolloid alone versus radiocolloid plus blue dye; thus, the study concluded that blue dye was not necessary for the identification of positive SLNs [20]. In a separate retrospective study with a cohort of 215 melanoma patients by Hu et al., only radiocolloid was used to identify SLNs, and the outcome compared with the addition of blue dye studies showed no difference [21]. Thus, the authors concluded that experienced surgeons could perform a SLN biopsy accurately with radioisotope tracer without the blue dye. In a phase 3 Technetium-99m-labeled Tilmanocept (Lymphoseek^®^) melanoma SLN clinical study, it was found that Technetium-99m-labeled Tilmanocept reached the prespecified primary concordance endpoint, identifying 98.7% of blue nodes. More importantly, it identified more SLNs in more patients and detected more melanoma-containing nodes than blue dye [22]. Since blue dye itself may not contribute significantly to the identification of SLNs and its association with increased cost and prolonged skin staining [23] as well as potential, albeit rare, anaphylactic side effects [24,25], the use of blue dye may not be warranted for identifying SLNs. The argument that it enhances visualization to show trainees the blue-stained lymphatics is appropriate. However, for experienced surgeons, radioactivity using a gamma probe is more efficient and reliable, as documented in the phase 3 Tilmanocept study [22], compared with the blue dye. There is an ongoing debate regarding removing lymph nodes with radioactivity below the highest count to prevent missing nodes with lower counts that might contain micrometastases. The current practice, supported by several studies, adheres to the “10% rule” [19], which suggests that nodes showing at least 10% of the radioactivity of the highest count node may contain micrometastases [20].

Sondak and Zager have carefully defined the false negative rate (FNR) as the ratio of false-negative results to the total number of positive lymph nodes (false negatives plus true positives); thus, the false negative rate has been reported in the range of 6–21% [26]. In the Sunbelt Melanoma Trial, as described by Scoggins and colleagues [27], 19.8% of participants (486 out of 2451) had a positive lymph node. The negative predictive value of the SLN biopsy was calculated to be 97.0%, based on 1906 true negative results out of 1965 total negative findings (false negatives plus true negatives). Additionally, the FNR of the biopsy was found to be 10.8%, computed as 59 false negatives divided by the sum of 59 false negatives and 486 true positives. In several large series of melanoma patients undergoing SLN surgery, Sondak and Zager reported that the false-negative rate ranged from 5.6 to 21% [26]. Valsecchi and colleagues conducted the most extensive meta-analysis on SLN biopsy to date [28], incorporating data from 71 studies totaling more than 25,000 patients. In this comprehensive analysis, the FNR varied widely, from 0% to 34%, with an average weighted rate of 12.5%. These differences in FNRs may be related to several factors; preoperative imaging techniques, differences in study methods, surgical methods during the operation, pathological examination, duration of follow-up, and the demographics of the patient population may influence the FNR. Therefore, while the SLN biopsy is an important diagnostic tool for melanoma, its FNR can fluctuate, emphasizing the need for surgeons and patients to be aware of these differences. In this review article, we will address these factors to maximize the identification rate and minimize the FNR of SLN biopsy for melanoma.

## 2. Techniques for Preoperative Identification of SLNs 

**Figure 1:**
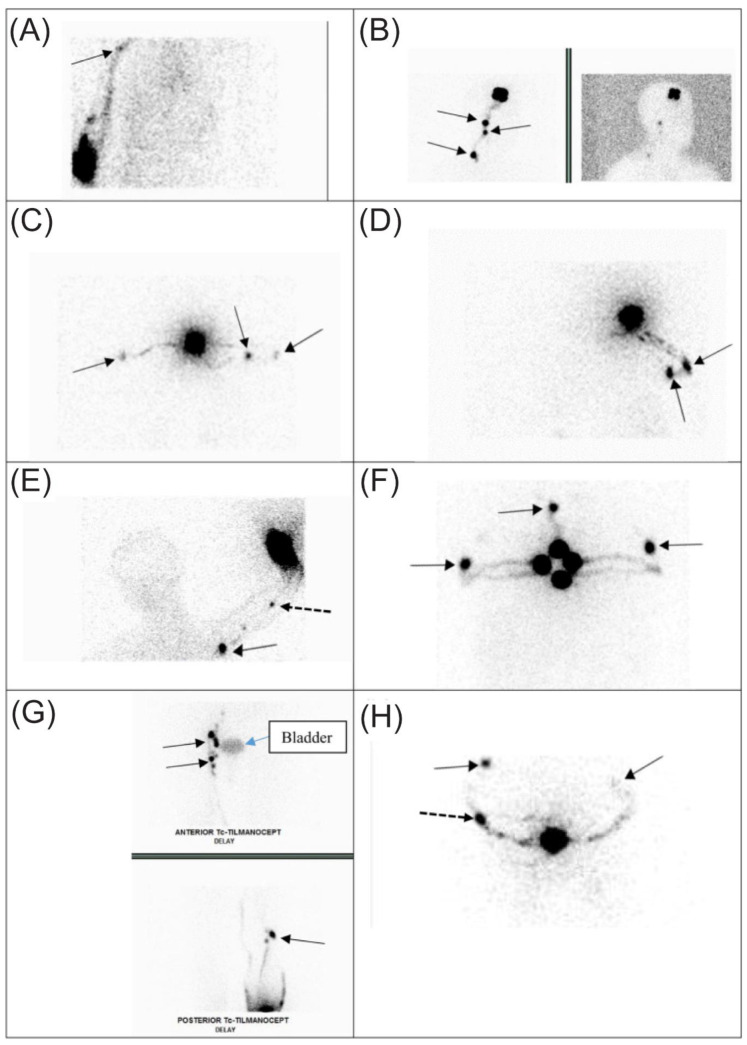
Preoperative lymphoscintigraphy demonstrates varying lymphatic channel patterns in patients with primary melanoma using Technetium-99m-labeled Tilmanocept (Lymphoseek^®^). Straight arrow = SLN; dashed arrow = in-transit SLN. (**A**) Drainage of a single channel from the right upper arm leading to one SLN in the right axilla. (**B**) Drainage of a single channel from a right parietal scalp lesion to multiple contiguous nodes in the right neck. (**C**) Confluent right channels drain from the upper back to two SLNs in the right axilla, and a single channel drains to a single SLN in the left axilla. (**D**) Multiple channels from the right upper back drain to two SLNs in the right axilla. (**E**) Multiple channels from the left proximal forearm lead to a single epitrochlear SLN and a single SLN in the left axilla. (**F**) Multiple drainage channels from a lesion in the anterior chest wall to SLNs in both axillae and one single SLN in the supraclavicular node at the suprasternal notch. (**G**) Multiple channels from the right lower extremity drain to multiple SLNs in the pelvic, femoral, and popliteal basin. Bladder activity is present in the image. (**H**) Multiple channels from the midline back draining to multiple SLNs in different basins, one in the right and one in the left axilla, and a single lateral left upper back in-transit SLN. The figure and legend are reproduced with permission from Leong, SP [16], *Clinical and Experimental Metastasis*, published by Springer Nature, 2021.

Since primary melanoma may arise from any site of the body, SLNs can be located in various nodal basins, such as the popliteal and inguinal basins for lower extremity melanomas, the epitrochlear and axillary basins for upper extremity melanomas, and multiple areas like the lower neck and bilateral axillae and groins for melanomas originating on the trunk. Also, occasional in-transit SLNs may be encountered. Therefore, a preoperative lymphoscintigraphy is mandatory to accurately map the nodal basins related to the primary melanoma site. Figure 1 illustrates the various lymphatic drainage patterns in different nodal basins.

We have described a recently developed radiotracer, Technetium-99m-labeled Tilmanocept (Lymphoseek^®^), which targets mannose receptors on macrophages within lymph nodes, leading to improved SLN detection. This tracer has been proven to be effective in identifying more SLNs and positive SLNs in melanoma, breast, head, and neck cancer patients compared to Lymphazurin, earning FDA approval for clinical use [16]. Additionally, other tracers like indocyanine green (ICG) [29] and superparamagnetic iron oxide [30,31] have been developed, with ICG being particularly useful during surgical procedures. However, Technetium-99m sulfur colloid and Tilmanocept remain the standard tracers for preoperative lymphoscintigraphy in melanoma [16]. At least 99% of the time, we can identify at least one SLN in at least one nodal basin. Once the SLNs are identified, the nuclear medicine physician marks them on the skin as targets for the surgeon to resect. Although infrequently, if there are two simultaneous primary sites, a lymphoscintigraphy will be performed sequentially so that nodal basins and SLNs can be noted precisely from each primary melanoma site. The lymphoscintigraphy is followed with SPECT/CT scans to enhance SLN localization [32,33]. Once the basin or basins of the SLNs are idenditfied and marked, the patient proceeds with a wide local excision of the primary site and SLN biopsy. In cases where the primary site has already been excised, which occurs in less than 5% of cases, a delayed SLN biopsy will be performed. Surgeons habitually communicate with nuclear medicine physicians regarding the exact location of the SLNs.

## 3. Techniques for Intraoperative Identification of SLNs

In a previous study [34] with melanoma databases from the California Pacific Medical Center and Moffitt Cancer Center in Tampa consisting of 564 melanoma patients (average age 60.3 years, 62% male) who underwent preoperative lymphoscintigraphy, at least one SLN was detected for each. The primary melanoma locations were varied: 27% were in the head/neck region, 33% on the trunk, 21% on the upper extremity, and 19% on the lower extremity. Among these, multiple draining basins were noted in 36.5% of head/neck, 36.4% of trunk, 13% for upper extremity sites, and 27.4% for lower extremity primary sites. On average, three SLNs (range 1–18) were identified and removed per patient. A total of 78% of patients had more than one SLN detected using Technetium-99m-labeled Tilmanocept. In a detailed analysis, the likelihood of identifying more than one SLN was significantly linked to factors such as age, Breslow depth, tumor location, and higher AJCC tumor stage. Out of the total study population, 17.7% (100/564) had a positive SLN. Overall, 145 positive SLNs were identified from a total of 1812 SLNs, resulting in a positive SLN rate of 8%. Factors such as younger age, increased Breslow depth, mitosis rate, higher AJCC tumor stage, presence of ulceration, and angiolymphatic invasion were significantly associated with positive SLN status.

The MSLT-II study indicated that CLND does not provide a therapeutic advantage over observation after a positive SLN biopsy. However, the status of the SLN remains a crucial indicator of prognosis for patients with primary melanoma [4,34], underscoring the importance of precise SLN identification to minimize the occurrence of missed positive SLNs.

Although the intraoperative handheld gamma probe is widely used to detect SLNs, it has several limitations: (1) its effectiveness largely depends on the operator’s skill; (2) small SLNs may escape detection from the gamma probe as it cannot cover the entire 360-degree spectrum of the operative field; (3) the SLNs may be at a different depth of the Neoprobe^®^ during roaming, especially in the axilla with a wide range of depth up to more than 6 cm; and (4) SLNs near the injection area may not be detected due to the “shine through” phenomenon. These factors may result in an increased FNR. Vidal-Sicart et al. have documented that an intraoperative gamma camera has been immensely helpful in complex melanoma cases [35]. Therefore, we wanted to test the utility of the intraoperative gamma camera Sentinella (Oncovision S.A., Valencia, Spain; https://oncovision.com/sentinella/, accessed on 1 June 2024) and determine if it would enhance the identification of SLNs in addition to the contribution of the Neoprobe^®^. Sentinella detects SLNs in the operating room, simultaneously combining the gamma and optical images in real-time.

**Figure 2:**
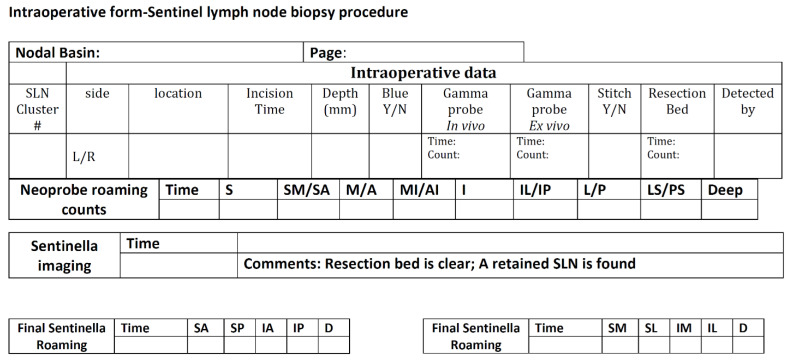
Intraoperative sterile data sheets are used to record the characteristics of each SLN cluster as # 1, 2 and etc. from the corresponding nodal basin to be dissected ex vivo after surgery (see Figure 3). Each page number is equivalent to the number of the cluster. When the resection bed and the Neoprobe^®^ roaming counts (eight positions of a clock) are higher over the background, additional exploration for the next cluster will be performed on a separate page. S = superior, SM/SA = superior medial/superior anterior, M/A = medial/anterior, MI/AI = medial inferior/anterior inferior, I = inferior, IL/IP = inferior lateral/inferior posterior, L/P = lateral/posterior, LS/PS = lateral superior/posterior superior, and Deep or D = over the center of the surgical bed. Roaming records by Neoprobe^®^ (8 positions of the clock) and Sentinella (4 positions of the clock) are included; SA = superior anterior, SP = superior posterior, IA = inferior anterior, IP = inferior posterior, and D = deep (resection bed); SM = superior medial, SL = superior lateral, IM = inferior medial, and IL = inferior lateral (New data).

In the operative room, the surgeon usually first makes a wide local excision of the melanoma, as Orme and Moncrieff [32] described in a Special Issue on Contemporary Surgical Management of Melanoma. General anesthesia is given almost 100% of the time. Most of the time (98%), SLN biopsy is an outpatient procedure. With the marking of the SLN by the radiologist in the appropriate nodal basin, the surgeon targets the marking using a handheld gamma probe, the Neoprobe^®^ Gamma Detection System with Bluetooth wireless technology (https://www.somatechnology.com/pdfFiles/Neoprobe-GDS-Gamma-Detection-System.pdf, accessed on 1 June 2024). The resection of each SLN cluster is recorded on a sterile record sheet (Figure 2). Each cluster could contain one or more SLNs as well as non-SLNs. Additional clusters are resected sequentially. Following the removal of each cluster, gamma probe roaming can be performed for eight positions of the clock, as shown in Figure 2 (New Data).

When the Neoprobe^®^ roaming counts show background readings with single or low double digits, a gamma camera such as Sentinella (Figure 4) can be used to scan the field to ensure that the operative field retains no SLNs. Using this approach, we have completed a prospective study to assess the utility of Sentinella to detect any remaining SLNs [36]. Preoperative imaging for 100 patients revealed 138 SLN basins, and traditional surgical methods by hand-held Neoprobe^®^ identified 306 SLNs. The Sentinella identified 89 extra SLNs in 54 patients, an increase of 23% [95% confidence interval (CI) 18–27%]. Among these additional SLNs, four harbored micrometastasis in four patients. Notably, Sentinella detected tumor-positive SLN in two of these patients, who otherwise were negative for a SLN biopsy, thus, preventing two potential false-negative outcomes. Consequently, the hypothesis that the PGC would not identify additional positive SLNs was disproven (*p* = 0.000). The overall positive SLN rate was 9.9% (39 out of 395, 95% CI 6–12), and the overall patient positive rate increased from 25% to 27% (27 out of 100) using Sentinella. In summary, intraoperative imaging with the Sentinella camera significantly improved SLN detection over traditional methods using a gamma probe alone. This enhancement in detection led to the upstaging of two patients being reclassified from negative to positive SLN status, thus averting two false-negative cases [36]. In a separate study, Sentinella has been found to detect more SLNs in breast cancer than the gamma camera in a group of 144 breast cancer patients undergoing SLN biopsy [37]. As mentioned above, there are multiple disadvantages to Neoprobe^®^. Thus, the Sentinella can pick up SLNs, otherwise missed by Neoprobe^®^ via its bird’s eye view of the operative field.

This improved detection method has prognostic value and plays a significant role in determining adjuvant systemic immunotherapy like pembrolizumab or nivolumab or targeted therapy with dabrafenib and trametinib. Therefore, accurate SLN biopsy and staging are essential for providing appropriate and effective adjuvant treatment to melanoma patients.

As mentioned above, a preoperative lymphoscintigraphy is mandatory to determine the location of the SLN basins, particularly in the mid-scalp, mid-neck, and mid trunk both anteriorly and posteriorly, as the midline location of the primary melanoma may result in bilateral nodal basins in the neck, axilla, and groin (Figure 1). Further, in the forearm, drainage may involve the epitrochlear nodal basin in about 5% of the cases in addition to the axillary basin. In a previous study by Miranda et al. [38], of 499 patients with lower extremity melanoma undergoing SLN biopsy, 356 had melanoma located below the knee (71%), and 143 (29%) had melanoma above the knee. Among those with below-the-knee melanoma, the node-positivity rates were distributed as follows: 23% (sixty-three out of two-hundred and seventy-one patients) were positive in the superficial inguinal basins, 0% were positive in the deep inguinal basins (zero out of three patients), and 50% were positive in the popliteal basins (one out of two patients). For those with above-the-knee melanoma, the positivity rates were 21% (twenty-four out of one-hundred and thirteen patients) in the superficial inguinal basins, 33% (one out of three patients) in the deep inguinal basins, and 0% in the popliteal basins, where no data were available due to the absence of cases involving this basin. Notably, the study found that no patients with a negative superficial inguinal SLN had a positive deep inguinal SLN upon final pathologic evaluation, indicating that a negative finding in superficial inguinal nodes strongly suggests the absence of metastasis in deeper inguinal nodes. From our Sentinel Lymph Node Working Group melanoma database (www.snoffoundation.org), we have found that there is no significant clinical benefit to resecting pelvic SLN biopsy in the setting of a negative inguinofemoral SLN biopsy based on the evaluation of 2476 cases of lower extremity and trunk melanomas [39]. This result supports the recommendation of not exploring the pelvis basin even if the lymphoscintigraphy shows the presence of pelvic SLNs. With the demonstration that CLND may show no clinical benefit in the MSLT-II study [4], lymph node dissection has been deferred in most cases when the tumor burden in the SLN is minimal, certainly less than 0.6 mm, as noted in the MSLT-II study. Patients with recorded pelvic SLN(s) will be followed, and if enlarged pelvic lymph nodes are found during a follow-up imaging study, systemic treatment may be given, consisting of checkpoint inhibition immunotherapy or targeted therapy. If pelvic lymph node dissection is indicated, robot pelvic lymph node dissection is used to resect the pelvic or external iliac lymph nodes [40,41].

## 4. The Synergistic Effect of Neoprobe^®^ and Sentinella (New Data)

Neoprobe^®^ can easily detect a single SLN. However, there are are several reasons as mentioned above that Neoprobe^®^ may not be able to detect a SLN. Therefore, imaging the nodal basin with the Sentinella provides a comprehensive bird’s eye view of the basin. A negative reading by the Sentinella is an excellent confirmation that there are no retained SLNs. On the other hand, if Sentinella shows a focal activity, the Neoprobe^®^ can be directed to the target area to localize the SLN to be resected. The bed of the nodal basin should be imaged again by Sentinella to ensure that the SLN found by Sentienella was properly resected.

## 5. Pitfalls of Identification of SLNs by Sentinella (New Data)

When an SLN has a strong focal activity, Sentinella detects it as a bright circular image (Figure 5A). An example of the surgical background with no retained SLNs after all the SLNs have been removed is depicted in Figure 5B. When the focal activity is weak (Figure 5C), it may represent channels versus an SLN with lower radioactivity. Thus, the Neoprobe^®^ is useful here to determine its radioactive count; if it is low or the count is less than 10% of the hottest SLN, it may be left behind and not be resected. When a strong and a weak SLN are adjacent, there is a sun and moon effect, when the bright SLN (sun) will render the weak SLN (moon) not detectable (Figure 5D). When the bright SLN is removed, the weak SLN becomes apparent (Figure 5E). In the right lower axilla, shine-through activity as a diffuse image with increased radioactivity may come from the liver (Figure 5F,G). In both the right and left inguinofemoral nodal basins, a diffuse shine-through activity may be noted from the bladder (Figure 5H,I). For this reason, a Foley catheter helps empty the bladder to minimize the shine-through effect. When the primary melanoma site, such as in the deltoid, lateral clavicular, and upper lateral chest wall, is excised, some residual radioactivity may still have a shine-through effect (Figure 5J,K). For this reason, the primary site should be excised before the SLN biopsy. Occasionally, the primary site has already been excised at the time of referral for a delayed SLN biopsy, and it would be challenging to avoid the shine-through effect. We have used a lead shield to block the shine-through effect of the primary injection site. Therefore, it is advisable that the primary site should be widely excised before the SLN biopsy. The pelvic SLN(s) will be detected by Sentinella, and during roaming, the pelvic SLN(s) will change positions as the Sentinella camera imaging goes around the four positions of the clock; we term such a change of position the “Galileo” effect.

## 6. Intraoperative Localization of Nerves in Different Sites for SLNB (New Data)

Despite the lesser risks of lymphedema, pain, and nerve damage associated with SLNBs compared to CLND [8,9], significant nerves are still at risk during SLN biopsy across various nodal basins. Examples include the spinal accessory nerve in the neck’s posterior triangle; the thoracodorsal and long thoracic nerve in the axillary area; the ulnar, median, and radial nerves in the deep epitrochlear region; the femoral nerve in the groin area; and the sciatic nerve in the popliteal fossa. Detecting and meticulously safeguarding these nerves is vital when performing lymph node dissections in these areas. Due to the narrower surgical area in SLN biopsy, locating these nerves can be more challenging. With its adaptable stimulation settings, the Checkpoint Nerve Stimulator (https://checkpointsurgical.com/nerve-care-products/protect-and-assess/checkpoint-9094, accessed on 1 June 2024) has shown exceptional utility in identifying these nerves before they are visually detected. A balanced, biphasic stimulation waveform permits ongoing stimulation of the tissue without reducing muscle response or risking nerve damage over time. This capacity for consistent stimulation allows for the continuous verification of nerve functionality, aiding in nerve preservation throughout the surgical procedure, particularly during complex dissections. Figure 6 shows the identification of the spinal accessory nerve in the supraclavicular basin, thoracodorsal nerve/long thoracic nerve in the axilla, femoral nerve in the proximal femoral basin, and sciatic nerve in the popliteal basin during SLN biopsy or lymph node dissection. In addition, videos accompanying this review article show these nerves upon stimulation by the Checkpoint Nerve Stimulator (Supplementary Video S1).

In conclusion, for melanoma lymph node surgeries—including SLN biopsy and CLND in the neck, axillary, epitrochlear, inguinal, and popliteal areas—it is our practice to always use a Checkpoint Nerve Stimulator for nerve verification to prevent injury. This is particularly crucial in SLN biopsy, where the smaller surgical field makes nerve identification challenging. Identifying the location of these nerves and avoiding injury to them are critical, as damage to these nerves can have severe consequences. 

## 7. Ex Vivo Dissection of SLN Clusters into SLNs and Non-SLNs Using the 10% Rule (New Data)

In a previous study by Rios-Cantu et al. [42], 291 adult melanoma patients underwent CLND following a positive SLNB. The 5-year disease-free survival and 5-year overall survival were compared between patients with additional positive lymph nodes and those without additional nodal involvement post-CLND. We found a significant difference in survival outcomes based on CLND status. For patients without additional positive lymph nodes, the 5-year disease-free survival (DFS) rate was 55% (95% CI 49–62%), while those with positive nodes post-CLND had a significantly lower DFS of 14% (95% CI 8–26%), as determined by the log-rank test (*p* < 0.0001). Similarly, the median DFS was notably longer in the negative CLND group at 7.4 years (95% CI 4.4–15+ years) compared to only 1.2 years (95% CI 1.0–1.8 years) in the positive CLND group. Regarding overall survival, the results were equally stark; patients with negative CLND had a 5-year overall survival rate of 67% (95% CI 61–74%), whereas those with additional positive nodes during CLND had a 5-year overall survival (OS) of 38% (95% CI 28–52%), with statistical significance again noted on the log-rank test (*p* < 0.0001). The median OS also reflected this trend, with 12.1 years (95% CI 9.3–15+ years) in the negative CLND group versus 2.5 years (95% CI 2.2–5.7 years) in the positive CLND group. This study supports the hypothesis that the progression of melanoma from the SLN to non-SLN compartments is orderly and indicative of worsening disease status, confirming the biological distinction between these compartments. The SLN acts as a critical gateway for further metastatic spread, underscoring its prognostic significance in melanoma. These findings highlight the importance of SLN status in melanoma management and suggest that additional positive nodes during CLND can significantly predict poorer outcomes, thus providing crucial information for both prognostic assessment and therapeutic planning. Other studies [43,44,45,46,47,48] have also shown that the non-SLN compartment carries a poorer prognosis. Therefore, it is critical to divide the specimens from the SLN biopsy to be submitted to pathology for careful examination of the SLN and non-SLN rather than submitting the entire specimen to pathology without ex vivo dissection. Based on the 10% rule [49], we have championed and adopted the ex vivo dissection technique for SLN biopsy specimens.

**Figure 3:**
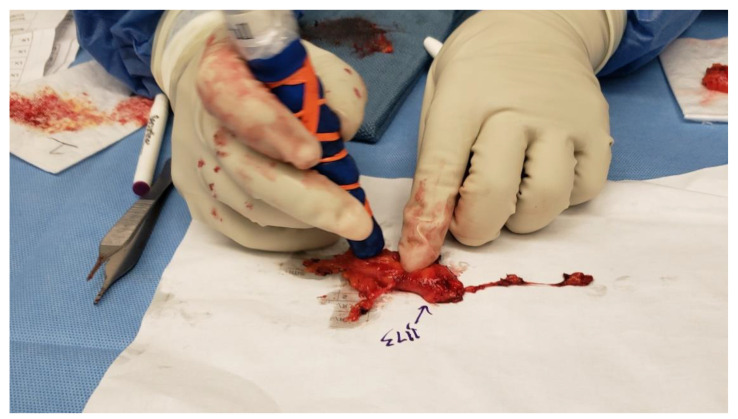
Ex vivo dissection of SLN biopsy specimens by the surgeon with ex vivo count for each specimen dissected to determine SLNs versus non-SLNs based on the 10% rule.

**Figure 4:**
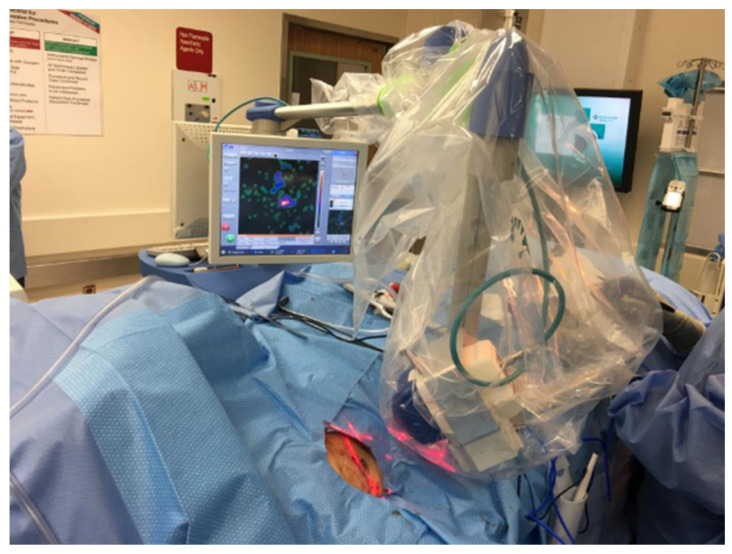
The Sentinella was draped sterilely and placed above the operative field after conventional surgery with Neoprobe^®^ identification of SLNs was completed. In this case, Sentinella found a retained SLN as a hotspot on the screen. This was confirmed with the Neoprobe^®^ and was subsequently removed. The figure and legend are reproduced with permission from Leong, SP [36]. Figure 1a of *Annals of Surgical Oncology* based on the http://creativecommons.org/licenses/by/4.0/, accessed on 1 June 2024.

**Figure 5:**
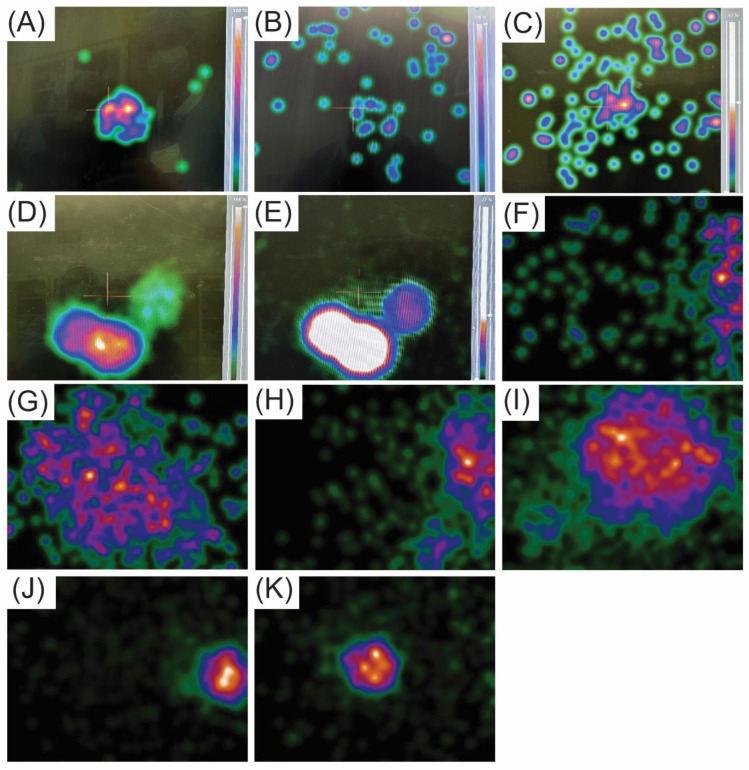
Identification of melanoma SLNs by Sentinella. (**A**) An example of when an SLN exhibits strong focal activity is when the Sentinella detects it as a bright circular image with the scale showing 100% level. Please ignore the reflections of the wall materials in the OR in the background. (**B**) The surgical background with no retained SLNs after all the SLNs have been removed. (**C**) An example of when the focal activity is weak. It may indicate channels rather than an SLN with lower radioactivity, as its radioactivity is enhanced when the scale is dropped to about 50%. In this situation, the Neoprobe^®^ is useful for measuring the radioactive count of a weak SLN. If the count is low or less than 10% of that of the hottest SLN, the weaker SLN may be left unresected. (**D**) When a strong and weak SLN is adjacent, a sun and moon effect occur, where the bright SLN (sun) obscures the weak SLN (moon), making it undetectable as the scale shows 100%. (**E**) Once the bright SLN is dimmed by lowering the scale to less than 50%, the weak SLN becomes visible. Following the resection of the SLN in the right axilla, the background is negative except for “some radioactivity” in the right corner near the edge of the screen (**F**). When the Sentinella was directed inferiorly to follow the site with increased activity, the shine-through effect of the liver was appreciated (**G**). Following the SLN biopsy of the right groin, radioactivity is noted by Sentinella on the right of the screen (**H**). The shine-through effect of the bladder is appreciated when Sentinella is directed over the increased radioactivity (**I**). After the resection of the SLN in in the right axilla (**J**), the bright focal activity is from the injection site with retained radioactivity following primary melanoma re-excision (**K**). No scale is shown from image (**F**) through (**K**), but all the scales are 100% except 50% in (**E**).

**Figure 6:**
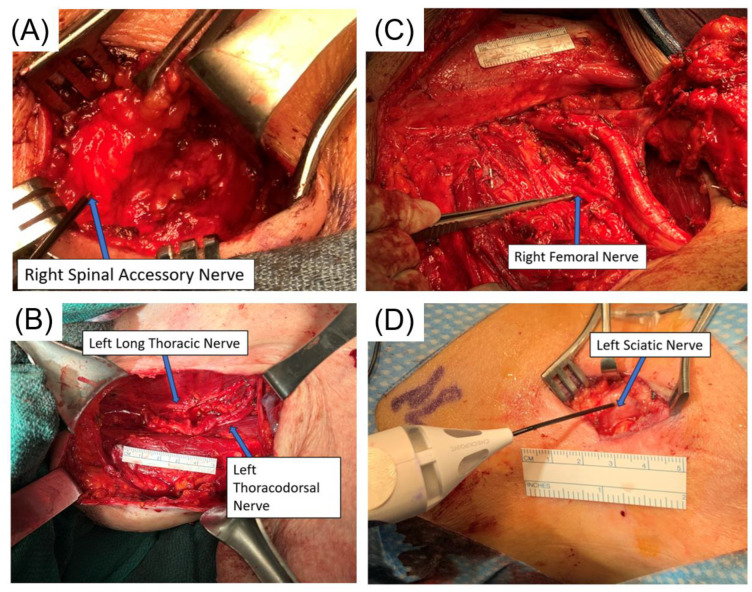
Identification of the spinal accessory nerve in the supraclavicular basin, thoracodorsal nerve and long thoracic nerve in the axilla, femoral nerve during inguinofemoral lymph node dissection, and sciatic nerve in the popliteal basin during SLNB. (**A**) Right supraclavicular selective SLN dissection showing the spinal accessory nerve being identified by the Checkpoint Nerve Stimulator; note that the nerve is embedded in the fatty tissue, which is not easily identified without the Stimulator. (**B**) Left axillary lymph node dissection showing thoracodorsal nerve and long thoracic nerve by Checkpoint Nerve Stimulator. (**C**) Right inguinofemoral lymph node dissection showing the right femoral nerve being identified by the Checkpoint Nerve Stimulator. (**D**) Left popliteal selective SLN dissection showing the sciatic nerve being identified by Checkpoint Nerve Stimulator; note that the sciatic nerve is superficial just below the subcutaneous tissue. Each nerve in each figure is shown in the accompanying video (Supplementary Video S1) to demonstrate its function in exciting its innervated muscle(s).

**Figure 7:**
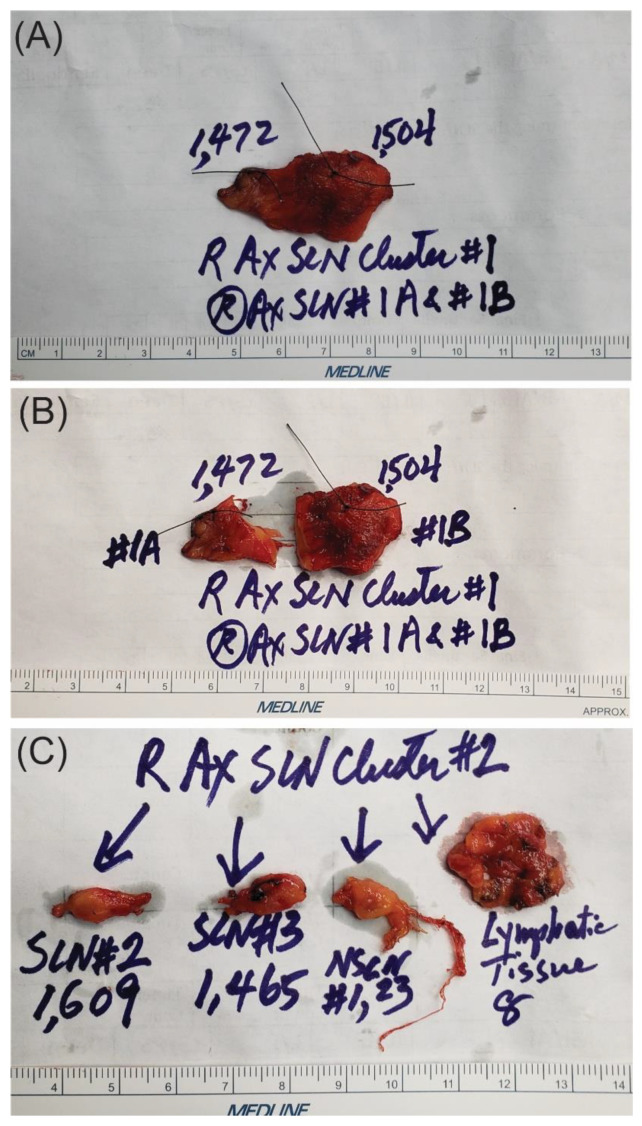
Ex vivo dissection of SLN clusters from the right axillary SLN biopsy specimens: (**A**) shows SLN cluster #1 was a large one with elevated count on each end of the lymph node; therefore, it was divided into SLN #1A of 1472 and SLN #1B of 1504 as noted in (**B**). The pathologist was provided with a note that SLN #1A and #1B were from SLN #1. Each silk stitch marks the highest count within the SLN. (**C**) shows that the right axillary SLN cluster #2 from another patient was indeed a cluster, and ex vivo dissection yielded SLN #2 (the number system was following SLN #1 from cluster #1) of 1609, SLN #3 of 1465, a non-SLN of 23 (less than 10% of the hottest lymph node) and lymphatic tissue of 8. A lymphatic channel to the right of the non-SLN in (**C**) has a diameter of about 100 microns. Each of the above specimens was submitted in a single formalin jar with a label to denote each lymph node’s characteristics, including its highest count (Figure 8).

**Figure 8:**
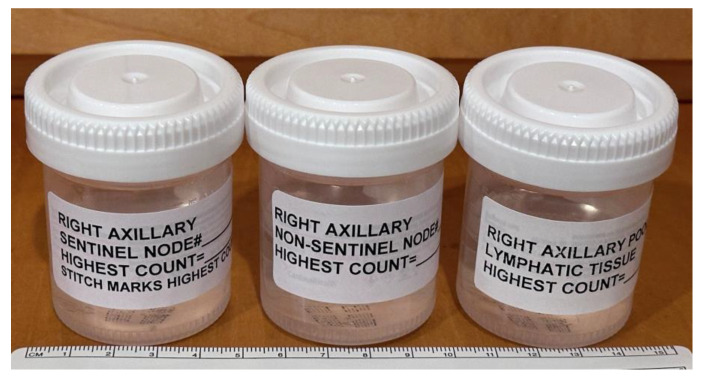
Formalin jars are used to submit each lymph node for pathological evaluation. Each formalin (10%) jar measures 6 cm in height and 4.5 cm in diameter. It contains only one specimen, an SLN, a non-SLN, or lymphatic tissue, which is to be submitted to pathology with its specific label and the radioactive count for histologic examination. The pathology report describes in detail the gross and microscopic features of each specimen within each jar.

**Figure 9:**
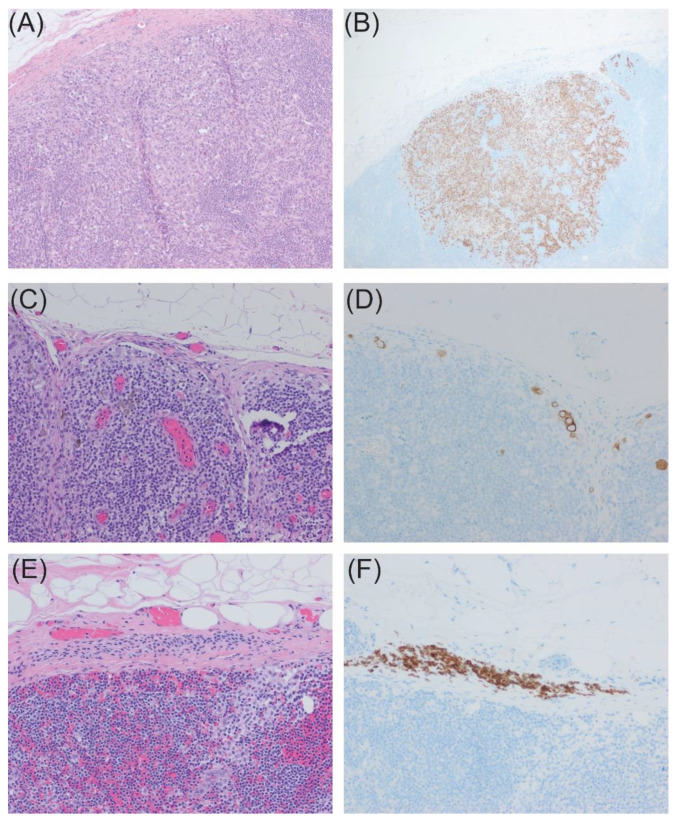
Histological evaluation of specimens from SLN biopsy: (**A**,**B**), SLN with 6 mm focus of metastatic melanoma identified by HE stain (100×) (**A**) and confirmed by Melan A immunohistochemical stain (**B**); (**C**,**D**), SLN with rare subcapsular melanoma cells seen on HE (200×) (**C**) with small tumor burden highlighted by Melan A immunohistochemical stain (**D**); (**E**,**F**), SLN with bland nevus cell identified within the capsule (200×) (**E**) also highlighted by Melan A immunohistochemical stain (**F**).

As noted above, when each SLN cluster was resected, it was kept on a Telfa and labeled as cluster #1, #2, #3, etc. Next, the clusters were transferred to a separate table with the recorded intraoperative forms (Figure 2). Each form and Telfa represented one cluster. Following the surgery, when the patient was transferred out of the operating room, the surgeon carefully dissected each cluster into lymphatic tissue, and SLNs versus non-SLNs (Figure 3 and Figure 7). Each dissected specimen was collected in a single and separate formalin jar with a specific label, such as lymphatic tissue (which was usually pooled), SLN #1, 2, 3, etc., with their corresponding radioactive counts as well as non-SLN #1, 2, 3, etc., with their radioactive counts based on the 10% rule as noted above. In this way, each lymph node is specifically labeled with no confusion between SLNs vs. non-SLNs, and each formalin jar is submitted to pathology separately (Figure 8). Each lymph node is examined using the pathological method as mentioned below regarding SLNs vs. non-SLNs (Figure 9). Figure 7A shows SLN cluster #1 from a left axillary SLN biopsy; upon ex vivo dissection, SLN cluster #1 was a large node with an elevated count on each end of the lymph node. Therefore, it was divided into SLN #1A of 1472 and SLN #1B of 1504 (Figure 7B). The pathologist was provided with a note that SLN #1A and #1B were from SLN #1. Figure 7C shows specimens from another patient, revealing that the right axillary SLN cluster #2 was indeed a cluster. The ex vivo dissection yielded SLN #2 of 1609 (the SLN number system is following SLN #1 from cluster #1), SLN #3 of 1465, a non-SLN of 23 (less than 10% of the hottest lymph node), and lymphatic tissue of 8. Each of the above specimens was submitted in a single formalin jar with a specific label to denote the characteristics of each lymph node with its highest count, as mentioned above (Figure 8).

## 8. Pathologic Examination of Sentinel and Non-SLNs (New Data)

Per recommendations of the College of American Pathologists (CAP), multiple levels through serially sliced SLNs increase the sensitivity of detecting microscopic melanoma metastasis. The entire lymph node should be submitted for histologic examination, with larger lymph nodes sliced at 2–3 mm intervals. However, submitting smaller lymph nodes whole (<5 mm) is satisfactory. Immunohistochemical stains additionally increase the sensitivity of detection of microscopic melanoma metastases and, therefore, should also be considered.

All harvested SLNs are fixed in 10% formalin following ex-vivo dissection in the operating room at our institution, serially sectioned at 2 mm intervals, and submitted in cassettes for paraffin embedding. Additionally, for lymph nodes designated as SLNs, unstained slides were cut at 40 µm intervals for the preparation of two flanking Hematoxylin and Eosin (HE) slides and four slides for immunohistochemical stains (Melan A, HMB45, S100, and negative control; Ventana Medical Systems (Tucson, AZ, USA), Benchmark Ultra Stainer) for histologic examination, which greatly aids in identifying lymph nodes with small tumor burdens. HE-stained slides are also prepared from the non-SLNs. Representative samples are shown in Figure 9. A black silk stitch to indicate the location of the highest radioactive account placed during ex-vivo dissection is very helpful for the pathologist to target this area of the SLN. 

## 9. Presentation of Melanoma Patients to the Melanoma Tumor Board (New Data)

When the primary melanoma meets the criteria of AJCC 8th edition [50] with a Breslow thickness of at least 1 mm, it is recommended that the patient should have an SLNB. The AJCC staging system has slight variations. The revised ASCO/SSO guidelines [51] recommend that new AJCC T1b patients undergo SLN biopsy, while T1a patients should not. In the United Kingdom, the recent consensus advises considering SLN biopsy for all T1b patients, particularly if there is a lymphovascular invasion or a mitotic rate greater than 2/mm^2^ [52]. The latest NCCN guidelines recommend SLN biopsy in patients with T1b lesions which are 0.8–1 mm thick or less than 0.8 mm thick with ulceration. The guidelines also suggest SLN biopsy for T1a melanoma patients with lymphovascular invasion, a mitotic rate of ≥2/mm^2^, or both. Another indication for SLNB is a partial biopsy of the original tumor with a positive deep margin close to the 0.8 mm threshold. The American Academy of Dermatology aligns with the NCCN recommendations, advising that SLN biopsy should be considered for T1a patients if high-risk histological features are present, the patient is younger than 40, or the primary tumor biopsy is inadequate or incomplete [53,54]. Our melanoma tumor board comprises members from the Departments of Pathology, Radiology, Dermatology, Surgical Oncology, Medical Oncology, Radiation Oncololgy, Nursing, and Genetic Counseling. Based on the final pathological results from the surgery, each patient’s case will be discussed, and treatment options will be rendered based on the consensus of the melanoma tumor board. Such treatment options will then be relayed to the patient by the physician involved in his or her care. Our melanoma database will record the diagnostic, pathologic, follow-up, and treatment outcomes for future reference and publications.

## 10. Conclusions and Future Perspectives

This review article emphasizes the importance of a multidisciplinary approach to treating melanoma patients when a primary melanoma is diagnosed; such patients should be referred to a melanoma center consisting of radiologists, pathologists, dermatologists, surgical oncologists, medical oncologists, and radiation oncologists. Our Pathology Department will confirm the patient’s primary diagnosis. Then, the patient undergoes a preoperative lymphoscintigraphy with SPEC/CT scans. Once the lymphoscintigraphy is used to define the basin or basins of the SLNs, the patient will proceed with a wide local excision of the primary site and SLN biopsy. All the specimens will be processed, and the slides will be read by our Pathology Department as previously described. The patient’s case will be presented at our weekly melanoma tumor board, attended by all the members of the above-stated departments. Follow-up or systemic treatment will be recommended based on the consensus of the melanoma tumor board. All the data throughout a patient’s treatment course from the time of diagnosis and eventual follow-up will be recorded in our melanoma database for compilation into reported studies for publication. Thus, melanoma represents a cancer type that requires multidisciplinary management in accordance with the above treatment procedures to enhance the best care of melanoma patients [55].

To lower the FNR of melanoma SLNBs, we have introduced and added the application of the intra-operative Sentinella gamma camera to survey the operative background following the traditional use of a gamma hand-held probe.

Our multidisciplinary melanoma diagnosis and treatment approaches should serve as a model for the treatment of other types of cancer. SLN status is still considered the most important prognostic factor for melanoma patients [56], although other biomarkers are being developed. The potential use of i31-GEP biomarkers in defining low-risk and high-risk melanoma patients for a positive SLN biopsy [57] is noteworthy, but future prospective studies are required to define their utility. Further, the microenvironment of sentinel lymph nodes is still poorly understood. Multiplex spatial imaging [58] and single cell transcriptomics [59] may allow us to define the cellular and molecular components of the melanoma sentinel lymph node microenvironment. These studies may lead us to the discovery of novel biomarkers relating to the progression of melanoma.

## Data Availability

The data presented in this study are available on request from the corresponding author.

## Supplementary Materials

The following supporting information can be downloaded at: https://www.mdpi.com/article/10.3390/cancers16152767/s1, Video S1: Videos show contraction of muscles upon nerve stimulation. (A) Contraction of the right trapezius muscle upon stimulation of the right spinal accessory nerve. (B) Contraction of the left latissimus dorsi muscle upon stimulation of the left thoracodorsal nerve. (C) Contraction of the right thigh muscles upon stimulation of the right femoral nerve. (D) Contraction of the left popliteal muscles upon stimulation of the left sciatic nerve.
