# Supplementary material for: Preoperative and Intraoperative Identification of Sentinel Lymph Nodes in Melanoma Surgery
Source: Cancers (Basel). 2024 Aug 5;16(15):2767. doi: 10.3390/cancers16152767 (PMC11312045; doi:10.3390/cancers16152767)
Supplement: Supplementary file 1 [file cancers-16-02767-s001.zip › Supplementary Video S1.pdf]

Supplementary Video S1. Videos show contraction of muscles upon nerve stimulation. (A) Contraction of the right trapezius muscle upon stimulation of the right spinal accessory nerve. (B) Contraction of the left latissimus dorsi muscle upon stimulation of the left thoracodorsal nerve. (C) Contraction of the right thigh muscles upon stimulation of the right femoral nerve. (D) Contraction of the left popliteal muscles upon stimulation of the left sciatic nerve.
